# European mature adults and elderly are moving closer to the Mediterranean diet—a longitudinal study, 2013–19

**DOI:** 10.1093/eurpub/ckac070

**Published:** 2022-06-17

**Authors:** Ricardo Alves, Julian Perelman

**Affiliations:** NOVA National School of Public Health, Universidade NOVA de Lisboa, Lisboa, Portugal; Comprehensive Health Research Center (CHRC), Escola Nacional de Saúde Pública, Universidade NOVA de Lisboa, Lisboa, Portugal; Public Health Research Center, NOVA National School of Public Health, Universidade NOVA de Lisboa, Lisboa, Portugal

## Abstract

**Background:**

The decreasing adherence in Mediterranean Diet (M.D.) during the last decades has been attributed to social, cultural and economic factors. However, recent efforts to improve dietary habits and the economic improvement might be reversing this trend. We analyze the changes in M.D. adherence between 2013 and 2019 among a sample of European mature adults and the elderly.

**Methods:**

Using data from the Survey of Health, Ageing and Retirement in Europe for adults over 50 years old, we designed a longitudinal cohort study with a sample of participants from waves 5 (2013) and 8 (2019/20). Logistic regressions were used to model the consumption of M.D. adherence as a function of the year. We then stratified the analyses by education, age and transitions in economic status, employment and self-perceived health.

**Results:**

There was in 2019/20 a significant increase in the M.D. adherence (10.8% vs. 14.3%, OR = 1.367, *P* < 0.01). The rise was mainly related to the decrease of meat and fish (38.4% vs. 30.5%, OR = 0.703, *P* < 0.01) and growth of legumes and eggs intake (36.3% vs. 41.8%, OR = 1.260 *P* < 0.01). The results were consistent in all European regions and most sociodemographic groups. Younger people with higher income and education had a greater rise in adherence.

**Conclusions:**

Our analysis shows a generalized growth in adherence to the M.D. across most socioeconomic subpopulations and countries in Europe, suggesting a shift to healthier diet patterns. The more noticeable increase among affluent, educated and healthy respondents, may further entrench dietary and health inequalities.

## Introduction

Adherence to the Mediterranean diet (M.D.) in Europe has been decreasing in most European countries since the mid-20th century,^1^ especially in Southern European countries and among younger people.^2–4^ The observed shift away from foods commonly linked to the M.D. is related to lower intakes of vegetables and fruits and increased ingestion of more processed foods with low nutrient density.^5^^,^^6^ As the M.D. is associated with better health outcomes and a lower risk of several non-communicable diseases,^7^^,^^8^ the long-term change to less healthy diet patterns might increase the burden of diet-related diseases in the future. Also, evidence from a meta-analysis showed that elderly individuals’ adherence to the M.D. was linked to prolonged survival, highlighting the importance of adopting or maintaining this diet for older people.^9^

Cultural, educational and economic factors have been linked to changes in populations’ diet patterns.^10–12^ In the last decades, changes to modern lifestyles linked to rapid urbanization and transformation of the work environment, dissemination of ready-to-eat outlets or technical changes in the food industry, and other barriers such as lack of time or skills to prepare healthier meals, have been pushing people away from the M.D.

More recently, studies have suggested a connection between the Great Recession and decreased adherence to the M.D.^13–15^ The higher price of some M.D. foods, making them less affordable for the worse-off, has been suggested as a possible explanation for this decrease. Although older people seem more resistant to dietary changes (keeping healthier food habits),^11^^,^^16^ they are among the most economically vulnerable people in Europe. In Italy (one of the most-affected countries during the last recession) the less-affluent elderly had a greater decrease in adherence to this diet.^14^

As the economic situation generally improved in Europe since the Great Recession (pre-Covid-19 pandemic), we could hypothesize a reversal in this trend, especially among those most hit by the economic downturn. Additionally, because of the overwhelming evidence linking poor diet patterns to an increasing number of chronic diseases^8^^,^^17^ during the last decade, national governments in Europe have been putting in place different strategies to improve dietary habits and reduce the burden of non-communicable diseases.^18^^,^^19^ The introduction of fiscal policies, new regulatory measures, limits to food marketing and healthy diet promotion campaigns may also play a role in the M.D.’s adherence. However, it is uncertain how effective these policies might have been among the elderly, who have shown to be less susceptible to the long-term trend changes in diet pattern.

Finally, it is essential to mention that older adults’ nutritional status is greatly influenced by physiological and health changes occurring at an older age.^20^^,^^21^ They are more vulnerable to nutritional deficiencies due to the deterioration of functions, such as appetite loss, dysphagia or changes in taste. Psychological factors such as depression and cognitive impairment common in these age groups could also significantly alter what and how much individuals eat.

We examine the changes between 2013 and 2019 in the consumption of selected food groups commonly linked to the M.D. among a sample of European elderly and analyze their geographic and socioeconomic patterning.

## Methods

### Study design and population

We performed a secondary analysis of the last two released versions of the SHARE project (‘Survey of Health, Ageing and Retirement in Europe’: wave 5 from 2013 and wave 8 from 2019/20). This survey is a cross-country research project collecting data from the European population aged over 50 years on health-, social- and economic-related topics. SHARE has data from the years 2004–20 featured in eight waves and allows the analysis of the same participants at different points in time. We therefore design this work as a longitudinal cohort study. This publication is based on preliminary SHARE wave 8 release 0 data. Therefore, the analyses, conclusions and results are preliminary.

We did not include data from waves 6 (2015) to 7 (2017) due to the few responses to the diet-related module in those waves. Indeed, this diet-related module was not performed in most countries in waves 6 and 7.

Our sample included responders from the following 13 European countries: Germany, Sweden, the Netherlands, Spain, Italy, France, Denmark, Switzerland, Belgium, Czech Republic, Luxembourg, Slovenia and Estonia. Thirteen European countries were left out of the analysis because the wave 8 data collection was interrupted by the Covid-19 pandemic restrictions.

To get a sample with the same participants in the two waves, we excluded individuals who did not participate in both wave 5 and wave 8 (*n* = 57 329), new respondents (those who were enrolled after wave 5, *n* = 8265) or people who deceased (*n* = 5266) between the two waves. The participants in our final sample present similar sociodemographic characteristics compared with the original data set (i.e. low educated 28.99% vs. 29.26%; poor economic status 27.76% vs. 29.34%; age group 50–60 18.24% vs. 22.82%; retired 66.34% vs. 63.19%).

### Outcome variable

M.D. data were assessed through the following survey questions [Possible responses: (i) Less than once a week; (ii) Once a week; (iii) Twice a week; (iv) three to six times a week; (v) Every day]. In a regular week, how often do you:

- have a serving of legumes, beans or eggs?
- eat meat, fish or poultry?
- consume a serving of fruits or vegetables?
- consume a serving of dairy products, such as a glass of milk, cheese in a sandwich, a cup of yoghurt or a can of high-protein supplement?

A binary index for M.D. adherence was constructed (value = 1 if following the diet; 0 not following) based on the daily consumption of fruits or vegetables (every day); and frequent intake of legumes, beans or eggs or meat, fish or poultry (three to six times a week).

Since the M.D. is based on eating habits from different locations in Spain, Italy and Greece in the 1960s, there is still some debate on the food groups included and frequency of consumption.^9^ Nevertheless, we based our index on the general consensus that indicates an M.D. consisting of a high intake of fruits, vegetables, wholegrain cereals, beans and nuts, a moderate intake of fish, poultry and eggs and low consumption of red meat.^7^^,^^22^

Our database does not differentiate red meat from other types of meat. Therefore, we tested if results were robust to marginal changes in meat consumption in our M.D. adherence index. We tested two indexes for M.D. adherence, based on lower weekly consumption of meat, fish or poultry (M.D1—Twice a week, M.D2—Once a week).

A similar index was used in a recent study using identical SHARE data, which showed a negative correlation of M.D. with the incidence of chronic illnesses and levels of depressive symptoms.^23^ Thus, the findings suggest that this M.D. index has predictive power for health outcomes that points in the same direction as other more detailed M.D. studies.

Individual food groups were also analyzed: we dichotomized the consumption of fruit and vegetables (1 = every day; 2 = ≤6 times/week); consumption of meat, fish or chicken (1 = every day; 2 = ≤6 times/week); consumption of legumes or eggs (1 = >3 times/week; 2 = ≤2 times/week); and consumption of dairy products (1 = every day; 2 = ≤6 times/week).

### Explanatory variables and covariates

The year of the interview (wave 5 = 2013 and wave 8 = 2019/20) was coded as a dichotomous variable. Covariates included gender (female/male), age as continuous variable (50–103 years old), country (14 countries’ fixed effects), educational level [primary education (9 years of education)/secondary education (12 years of education)/tertiary education (>12 years of education)], economic deprivation [household able to meet ends meet (with difficulty = poor/fairly easily = fair/easily = good)], employment (retired/employed or self-employed/permanently sick or disabled/homemaker/unemployed) and self-perceived health (good/fair/poor).

### Statistical analysis

Logistic regressions were used to model the consumption of M.D. adherence, meat/fish, fruit/vegetables, legumes/eggs and dairy products as function of the year. We performed stratified analyses by educational level and age groups and for transitions in economic status, employed and self-perceived health between 2013 and 2019/20. This analysis enables us to explore the possible differences in M.D. adherence according to transitions in economic status, employment or health. All regression models were controlled for gender, educational level, economic deprivation, employment, self-perceived health and country.

To check if the trend in M.D. adherence was not linked to the ageing of the population during the observation period (6/7 years), we performed a robustness check by running an additional stratified analysis by age: we measured the change in M.D. adherence within different people of the same age in waves 5 and 8, e.g. we compared the M.D. adherence among people aged 50–55 years old in waves 5 and 8. In other terms, we performed an analysis using the data as repeated cross-section instead of considering its longitudinal nature.

## Results

The same 22 804 participants were included in the 2013 and 2019/20 analyses (table 1). Between 2013 and 2019/20, the percentage of retired individuals rose from 57.1% to 75.7%.

**Table 1:** Baseline characteristics of participants for 2013 and 2019/20

Variables	2013 (%)	2019/20 (%)
Age groups		
50–60	7684 (33.7)	2261 (9.9)
61–70	8855 (38.8)	8508 (37.3)
71–80	5113 (22.4)	8083 (35.4)
>81	1152 (5.1)	3952 (17.1)
Educational level		
Primary	6,611 (28.9)	6611 (28.9)
Secondary	7,168 (31.4)	7168 (31.4)
Tertiary	9,025 (39.5)	9025 (39.5)
Economic status		
Poor	6925 (30.3)	5485 (24.1)
Fair	6608 (28.9)	7742 (33.9)
Good	9271 (40.6)	9576 (41.9)
Self-perceived health		
Poor	7239 (31.7)	8523 (37.3)
Fair	8765 (38.4)	8811 (38.6)
Good	6795 (29.8)	5470 (23.9)
Employment		
Retired	13 005 (57.1)	17 075 (75.7)
Employed	6984 (30.6)	3517 (15.6)
Permanently sick/disabled	680 (2.9)	490 (2.1)
Homemaker	1505 (6.6)	1200 (5.3)
Unemployed	630 (2.7)	259 (1.2)

**Figure 1:**
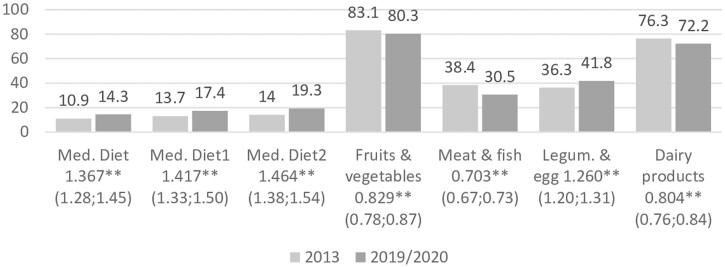
Adjusted percentage for M.D. adherence and consumption of key foods in 2019/2020 vs. 2013, and adjusted odds ratios (95% CI) for 2019/20 vs 2013 Note: Adjusted for gender, educational level, age, economic status, employment, self-perceived health and country. *p < 0.0.5; ** *p* < 0.01.

In 2019/20, there was an overall significant increase in the M.D. adherence (10.9% vs. 14.3%, OR = 1.367, *P* < 0.01; figure 1). We observed a similar trend even with marginal changes of meat consumption in our M.D. adherence index. When compared with 2013 there was in 2019/20 a significantly lower percentage of people who reported a daily consumption of fruits and vegetables (83.1% vs. 80.3%, OR = 0.829, *P* < 0.01), meat and fish (38.4% vs. 30.5%, OR = 0,703, *P* < 0.01) and dairy products (76.3% vs. 72.2%, OR = 0.804, *P* < 0.01). Inversely, the frequent consumption (>3 times/week) of legumes and eggs significantly increased (36.3% vs. 41.8%, OR = 1.260 *P* < 0.01) during the same period.

People with poor economic status had a lower increase in M.D. adherence (OR = 1.288, *P* < 0.01) when compared with the M.D. adherence increase of the higher economic status sub-group (OR = 1.501, *P* < 0.01; table 2). This trend is mainly the result of the greater decrease in meat and fish consumption among people with good economic status (OR = 0.624, *P* < 0.05). The groups of people who experienced an improvement in their economic state from 2013 to 2019/20 increased the M.D. adherence significantly (OR = 1.294, *P* < 0.01; table 2). We observed a similar greater adherence for the people whose economic situation worsened (OR = 1.391, *P* < 0.01). Unlike the first group, the consumption of fruits and vegetables (OR = 0.756, *P* < 0.01), meat and fish (OR = 0.784, *P* < 0.01) and dairy products (OR = 0.783, *P* < 0.01) fell significantly.

**Table 2:** Adjusted odds ratios (95% CI) for M.D. adherence and consumption of key foods in 2019/0 vs. 2013 according to educational level, age groups and economic status, employment and self-perceived health transitions

2013	2019/20	M.D. adherence	Fruits and vegetables	Meat and fish	Legumes and eggs	Dairy products
Economic status						
Good	Poor	1.391 (1.09; 1.77)^**^	0.756 (0.61; 0.92)^**^	0.784 (0.66; 0.92)^**^	1.387 (1.16; 1.64)^**^	0.783 (0.65; 0.93)^**^
Poor	Good	1.294 (1.08; 1.55)^**^	1.101 (0.95; 1.26)	0.927 (0.81; 1.05)	1.130 (0.99; 1.28)^*^	0.983 (0.86; 1.11)
Good	Good	1.501 (1.37; 1.63)^**^	0.869 (0.80; 0.93)^**^	0.624 (0.58; 0.66)^**^	1.270 (1.19; 1.34)^**^	0.776 (0.72; 0.82)^**^
Poor	Poor	1.288 (1.08; 1.53)^**^	0.861 (0.75; 0.97)^*^	0.939 (0.82; 1.06)	1.358 (1.20; 1.53)^**^	0.867 (0.77; 0.97)^**^
Employment						
Employed	Retired	1.119 (0.91; 1.37)	0.792 (0.66; 0.95)^**^	0.669 (0.57; 0.78)^**^	1.102 (0.95; 1.27)	0.736 (0.62; 0.86)^**^
Employed	Employed	1.427 (1.15; 1.77)^**^	0.916 (0.76; 1.09)	0.581 (0.49; 0.68)^**^	1.114 (0.96; 1.29)	0.720 (0.60; 0.85)^**^
Retired	Retired	1.380 (1.26; 1.51)^**^	0.742 (0.69; 0.79)^**^	0.755 (0.91; 1.37)^**^	1.233 (1.15; 1.31)^**^	0.855 (0.80; 0.91)^**^
Self-perceived health						
Good	Poor	1.307 (1.11; 1.53)^**^	0.749 (0.65; 0.85)^**^	0.660 (0.58; 0.74)^**^	1.255 (1.12; 1.40)^**^	0.772 (0.68; 0.87)^**^
Poor	Good	1.255 (1.01; 1.55)^*^	0.855 (0.72; 1.00)	0.846 (0.72; 0.98)^*^	1.389 (1.19; 1.61)^**^	0.831 (0.71; 0.96)^**^
Good	Good	1.429 (1.31; 1.55)^**^	0.778 (0.72; 0.83)^**^	0.633 (0.59; 0.67)^**^	1.206 (1.13; 1.28)^**^	0.789 (0.73; 0.84)^**^
Poor	Poor	1.228 (1.07; 1.41)^**^	0.901 (0.81; 0.99)^*^	0.852 (0.77; 0.93)^**^	1.287 (1.17; 1.41)^**^	0.806 (0.73; 0.88)^**^
Education level						
Primary		1.372 (1.23; 1.52)^**^	0.860 (0.78; 0.94)^**^	0.765 (0.70; 0.83)^**^	1.271 (1.17; 1.37)^**^	0.919 (0.84; 0.99)^*^
Secondary		1.284 (1.15; 1.43)^**^	0.866 (0.79; 0.94)^**^	0.727 (0.67; 0.78)^**^	1.278 (1.18; 1.37)^**^	0.843 (0.78; 0.90)^**^
Tertiary		1.437 (1.30; 1.57)^**^	0.922 (0.85; 0.99)^*^	0.645 (0.60; 0.68)^**^	1.243 (1.16; 1.32)^**^	0.804 (0.75; 0.86)^**^
Age groups						
50–60		1.413 (1.21; 1.64)^**^	0.993 (0.87; 1.12)	0.635 (0.56; 0.71)^**^	1.347 (1.20; 1.50)^**^	0.798 (0.71; 0.89)^**^
61–70		1.455 (1.32; 1.60)^**^	0.810 (0.74; 0.87)^**^	0.648 (0.60; 0.69)^**^	1.277 (1.19; 1.37)^**^	0.785 (0.73; 0.84)^**^
71–80		1.240 (1.10; 1.38)^**^	0.813 (0.73; 0.89)^**^	0.756 (0.69; 0.82)^**^	1.175 (1.08; 1.27)^**^	0.862 (0.78; 0.94)^**^
>81		1.212 (1.00; 1.45)^**^	0.809 (0.68; 0.95)^**^	0.918 (0.81; 1.03)	1.367 (1.20; 1.55)^**^	0.902 (0.78; 1.03)

Notes: Adjusted for gender, educational level, age, economic status, employment, self-perceived health and country.

* *P* < 0·05; **P < 0·01.

People who retired between 2013 and 2019/20 were the only ones without significant changes in M.D. adherence (OR = 1.119, *P* > 0.05). In contrast, people who did not retire had a significantly higher M.D. adherence in 2019/20 (OR = 1.427, *P* > 0.01).

Changes in self-perceived health led to similar changes in M.D. adherence. However, the group of people who stated being in a poorer health condition in 2019/20 had a greater significant decrease in the consumption of fruits and vegetables (OR = 0.749, *P* < 0.01), meat and fish (OR = 0.660 *P* < 0.01) and dairy products (OR = 0.772, *P* < 0.01).

The increase in adherence was slightly lower among people with secondary education and age groups above 70.

Results from our robustness check showed that between 2013 and 2019/20, the variation patterns in M.D. adherence was similar among most age groups (Supplementary appendix SA1).

Finally, the analysis stratified by country showed a significant increase in M.D. adherence in most European countries (Supplementary appendix SA2).

## Discussion

### Key findings

Our results show that M.D. adherence increased among mature adults and the elderly in Europe between 2013 and 2019. The trend was observed in all socioeconomic groups except for people who retired during this period. The change was mainly related to the growth in legumes, beans and eggs uptake and to a reduction of daily animal protein intake. Still, we observed a slight decrease in the daily consumption of fruits and vegetables.

The growth in M.D. adherence was more prevalent among younger age groups, with higher income and tertiary education, and overall better self-perceived health.

### Interpretation

Contrary to most evidence from the last decades,^1^^,^^4^^,^^14^^,^^24^ our data seem to indicate a shift to a diet pattern closer to the M.D. This increase was visible in almost all European countries, most notably in northern Europe, where this diet pattern has gradually become more common since the 1960s.^1^ Our results were obtained on very recent data, so that differences from findings reported in earlier studies may result from a real change in early trends.

The increase in M.D. adherence was felt more prominently among individuals in a comfortable economic situation. The difference is mainly related to the greater decrease in meat and fish consumption among the better-off. These findings suggest that the decline in these animal proteins is not connected to the food group’s affordability. Low-income people may already start from a lower point of meat and fish consumption due to the higher price. Therefore, they might be in a position where the reduction in this food group’s weekly intake is less likely to occur.

The economic status transition analysis supports this same proposition: individuals who had difficulties meeting ends meet and moved to a better financial status in 2019/20 did not decrease their meat and fish intake. Also, this subpopulation was the only one that had a small increase in vegetables and fruit consumption. In contrast, the group of people that experienced a worsening of the household economic situation had some of the sharpest reductions in vegetables, fruits, meat, fish and dairy daily intake. However, this was the group with the greatest increase in legumes and eggs consumption. Loss of purchasing power might have led to the replacement of more expensive foods (fruits/vegetables and meat/fish) with less costly ones (legumes/eggs).^25–27^

Interestingly, this presents a more nuanced picture of the household budget impact on M.D. adherence. It was a similar growth in these opposing subpopulations, i.e. people who experienced improvement vs. decline of household budget: while the first group consumed more vegetables and fruits in 2019/20, the second reduced meat and fish and increased legumes and beans intake.

Health status also seems to affect dietary patterns: people who claimed to be in good health significantly reduced meat and fish intake and increased their consumption of legumes and eggs in 2019/20, which explains why the M.D. adherence was greater in this subpopulation. Noticeably, the individuals whose health deteriorated had the highest reduction in vegetables, fruits, meat, fish and dairy daily intake. This finding is in line with the evidence that links poorer health and diet changes due to appetite loss, dysphagia and other illness symptoms,^20^^,^^21^^,^^28^ which may also result in disease-related malnutrition. Still, a reverse causation may also help explain this connection—for instance, when poorer diet habits ultimately lead the worsened health condition.

Our data suggest some effects of retirement on M.D. adherence. Unlike people who did not change employment status, our analysis shows no significant increase in M.D. adherence for responders who retired during the 6/7 years analysis period. The withdrawal from ones’ occupation or working life appears to have a more notable effect on the reduction of fruits and vegetable consumption. Contrary to other subgroups, no increase in legumes and eggs was observed for this period. The literature on this topic has produced mixed findings: on the one hand, healthy eating may increase because people eat out less and have more time to prepare and cook food at home.^29^ On the other hand, working people may be forced to retire due to health complications, leading to changes in diet habits linked to the new health condition.^30^^,^^31^ Moreover, retirement is commonly associated with income loss,^32^ which may also imply changes in what people eat.

Education level seems to play a role in M.D. adherence changes. The results show a slight gradient in meat and fish consumption with a greater reduction among the more educated, which leads to a more substantial growth of M.D. for people with tertiary education. The available evidence overwhelmingly suggests a greater adherence to the M.D. among the higher educated,^3^^,^^23^^,^^33^ and our findings hint at the continuous entrenchment of this healthy diet habits gap between high- vs. low-educated people.

The lower M.D. adherence among older age groups may be linked to this cohort’s socioeconomic characteristics: most senior people in Europe are probably retired, usually less educated, more often economically vulnerable and have a poorer health condition. In this study, we found all of these factors to have a significant weight in the M.D. adherence.

Finally, environmental concerns about the impact of meat consumption could also be presented as a plausible hypothesis for the observed decrease in daily meat consumption and subsequent growth of M.D. adherence. A recent systematic review regarding consumer attitudes towards environmental concerns of meat consumption found that consumers are increasingly aware of the overall impact of meat production and are willing to stop or significantly reduce meat intake.^34^ Although the authors emphasize that the proportion of people who changed their meat intake is still a small minority, they also point out that this trend is becoming more common, especially in some Western Europe.

### Policy implications

It is not clear from our findings that the economic recovery in Europe was a driving factor of the increase in M.D. adherence. Our inconclusive results add to the existing complex and often contradictory evidence on the effects of economic downturns and recoveries on eating behaviours.^14^^,^^35–38^

Nevertheless, the shift to a healthier diet pattern contrasts with the evidence from the beginning of the last decade that linked the economic crisis with a decrease in M.D. adherence among southern European countries. We can argue that the economic recovery enabled an environment in which other factors could more likely affect the overall M.D. adherence. For instance, the meat consumption reduction was least significant among the worse-off and mostly felt among people in a comfortable economic situation. Moving to healthier diet patterns may be easier if a reduced household budget does not constrain food choices. This could allow, for instance, the concerns around the long-term effects of unhealthy diets on health and the environmental impact of meat consumption to play a more significant part in dietary patterns.

Notably, our data point to a further increase in dietary habit inequalities within different economic, educational, health and age groups. It shows that our individual food choices are inherently bound to our social and economic position and the environment we currently live in. Hence, the urgent shift to healthier diet patterns may only be achieved by also changing the population’s welfare conditions as a whole. Failure to address these social and economic disparities will only deepen diet and health inequalities.

## Conclusions

Our findings suggest a generalized growth in M.D. adherence among mature adults and the elderly population in Europe. The reduction of animal protein consumption and rise of legumes intake were the main drivers for this shift to a healthier diet pattern. We could not find a clear link between the economic recovery in Europe and the observed changes. However, the better economic background may have enabled an environment in which health and environmental concerns could play a part in M.D. adherence.

Although the increase was consistent across most socioeconomic subpopulations, it was more evident among more affluent, educated and healthy responders, which may further entrench diet and health inequalities.

### Strengths and limitations

The data from the Share surveys allow a longitudinal analysis with large representative samples of European populations. Thus, it was possible to explore cross-national and cross-wave comparability, considering the multidimensional characteristics (sociodemographic, health risks and well-being) of the same respondents over a period of time.

As usual, when working with larger samples, questions regarding food consumption were not based on food diaries, the evidence of which has shown to provide greater precision and validity.^39^ Instead, our sample’s food intake was measured by relying on the participant’s memory and could be biased by social desirability or social approval.^40^ Nevertheless, we can argue that having a large representative sample of 13 European countries outweighs this limitation.

Our M.D. adherence index is not based on a comprehensive, validated Mediterranean score^5^ and does not differentiate the food groups’ quality or quantity. For instance, we do not know the amount of added sugars in these products, if the meat or fish consumed is processed, or how much ‘daily intake’ implies in terms of calories. Nevertheless, we constructed our index based on a similar one that showed a negative correlation of M.D. with the incidence of non-communicable diseases,^23^ suggesting a predictive power for health outcomes as demonstrated in other validated instruments for assessing this diet.

Finally, the change in M.D. adherence is likely to be related to changes in prices, in diet-related policies (e.g. health promotion interventions or taxes on unhealthy foods), or in public awareness (e.g. the social media have increasingly reported the consequences of dietary patterns on health and climate change). Yet, our data did not allow estimate the impact of such contextual factors, which we intended to capture, imperfectly, through country-fixed effects. Yet, our aim was not to identify causal mechanisms, which would require different data and analyses. Further research is expected on this topic.

## Supplementary data

Supplementary data are available at *EURPUB* online.

## Funding

R.A. is a PhD candidate funded by the Portuguese Foundation for Science and Technology—FCT [2021/06359/BD]. This institution had no role in the study design; collection, analysis and interpretation of data; the writing of the article; or the decision to submit the article for publication. This publication was also funded by the Portuguese Foundation for Science and Technology—FCT, IP national support through CHRC (UIDP/04923/2020).

*Conflicts of interest*: None of the authors report any conflicts of interest.

Key points

- M.D. adherence increased among mature adults and the elderly in Europe (2013–19).
- Reduction of animal protein consumption and rise of legumes as the main drivers for M.D. adherence growth.
- M.D. adherence increases more evident among affluent, educated and healthy responders.
- Our findings suggest an entrenchment of diet and health inequalities.

## Supplementary Material

ckac070_Supplementary_Data
