# Supplementary material for: European mature adults and elderly are moving closer to the Mediterranean diet—a longitudinal study, 2013–19
Source: Eur J Public Health. 2022 Jun 17;32(4):600–5. doi: 10.1093/eurpub/ckac070 (PMC9341670; doi:10.1093/eurpub/ckac070)
Supplement: ckac070_Supplementary_Data [file ckac070_supplementary_data.docx]

**Appendix A**

A1. Adjusted odds ratios (95% CI) for Mediterranean Diet adherence 2019/2020 v. 2013, stratified by age

|  | M.D. Adherence | Fruits & Vegetables | Meat & Fish | Legumes & Eggs | Dairy Products |
| --- | --- | --- | --- | --- | --- |
| All groups | 1.376**(1.31;1.44) | 0.883**(0.84;0.92) | 0.794**(0.77;0.81) | 1.253**(1.22;1.28) | 0.896**(0.87;0.92) |
| 50 - 55 | 2.033(0.88;4.66) | 3.124(0.93;10.39) | 0.631(0.30;1.30) | 1.149(0.59;2.23) | 0.702**(0.53;0.91) |
| 56 - 60 | 1.271(0.88;1.83) | 0.870*(0.84;0.92) | 0.798(0.60;1.05) | 1.056(0.81;1.08) | 0.797**(0.71;0.88) |
| 61 - 65 | 1.446**(1.28;1.62) | 0.766(0.56;1.04) | 0.649**(0.58;0.72) | 1.317**(1.19;1.45) | 0.814**(0.74;0.88) |
| 66 - 70 | 1.462**(1.30;1.63) | 0.891*(0.79;1.00) | 0.658**(0.59;0.72) | 1.180**(1.08;1.28) | 0.756**(0.68;0.83) |
| 71 - 75 | 1.296**(1.14;1.46) | 0.853**(0.76;0.94) | 0.661**(0.60;0.72) | 1.316**(1.21;1.43) | 0.906(0.80;1.01) |
| 76 - 80 | 1.219**(1.06;1.40) | 0.908(0.80;1.02) | 0.795**(0.71;0.88) | 1.259**(1.13;1.39) | 0.911(0.84;0.92) |
| 81 - 85 | 1.396**(1.18;1.65) | 0.911(0.78;1.05) | 0.874(0.76;1.0) | 1.376**(1.21;1.55) | 0.901(0.78;1.03) |
| 86 - 90 | 1.434**(1.11;1.85) | 1.179(0.92;1.42) | 0.836(0.68;1.02) | 1.238*(1.03;1.47) | 0.854(0.69;1.04) |
| >91 | 1.163(0.72;1.86) | 0.785(0.53;15) | 0.931(0.65;1.31) | 1.114(0.81;1.52) | 0.807(0.5;1.17) |

Adjusted for gender, educational level, age, economic status, employment, self-perceived health and country

* P<0·05 / ** P<0·01

A2. Unadjusted percentage for Mediterranean Diet adherence in 2019/2020 v. 2013, and adjusted odds ratios (95% CI) for 2019/2020 v. 2013, stratified by country and U.N. geoscheme

* P<0·05 / ** P<0·01
